# Development and Experimental Validation of a Physics-Based Digital Twin for Railway Freight Wagon Monitoring

**DOI:** 10.3390/s26020643

**Published:** 2026-01-18

**Authors:** Alessio Cascino, Leandro Nencioni, Laurens Lanzillo, Francesco Mazzeo, Salvatore Strano, Mario Terzo, Simone Delle Monache, Enrico Meli

**Affiliations:** 1 Department of Industrial Engineering, University of Florence, 50139 Florence, Italy; 2 Department of Mechanical Engineering, Politecnico di Milano, 20133 Milan, Italy; 3 Department of Industrial Engineering, University of Naples, 80138 Naples, Italy; 4 Mercitalia Intermodal SpA, 20151 Milano, Italy

**Keywords:** multibody simulation, digital twin, railway dynamics, articulated freight wagon, railway vehicle, experimental validation, high-fidelity modeling

## Abstract

The development of digital twins for railway freight vehicles represents a key step toward more efficient, data-driven maintenance and safety assessment. This study focuses on the creation of a digital twin of the T3000 articulated freight wagon, one of the most widespread intermodal transport solutions in Europe. Despite its relevance, the dynamic behavior of this vehicle type has been scarcely investigated so far in scientific literature. A dedicated onboard measurement layout was defined to enable comprehensive monitoring of vehicle dynamics and the interactions between adjacent wagons within the train. The experimental setup integrates inertial sensors and a 3D vision system, allowing for detailed analysis of both rigid-body and vibrational responses under real operating conditions. A high-fidelity multibody model of the articulated wagon was developed and tuned using the acquired data, achieving optimal agreement with experimental measurements in both straight and curved track segments. The resulting model constitutes a reliable and scalable digital twin of the T3000 wagon, suitable for predictive simulations and virtual testing. Future developments will focus on a deeper investigation of the buffer interaction through an additional experimental campaign, further extending the digital twin’s capability to represent the full dynamic behavior of articulated freight trains.

## 1. Introduction

The freight railway sector plays a critical role in the global economy, offering a cost-effective, sustainable, and high-capacity solution for the transportation of goods over medium and long distances. In many national and international contexts, the efficiency and reliability of freight wagons directly influence the competitiveness of entire industrial supply chains. As the demand for increased load capacity, operational flexibility, and interoperability grows, there is a pressing need for innovation in freight vehicle design and performance assessment. However, the development of freight wagons has historically lagged behind passenger vehicles in terms of research attention, particularly in the domain of dynamic behavior modeling. Addressing this gap is essential for improving safety, reducing maintenance costs, and supporting the adoption of more advanced technologies in freight operations. Multibody modelling has become an essential tool in the analysis, design, and optimization of railway vehicles and freight wagons, enabling detailed simulation of dynamic behaviors under various operational scenarios. Early and ongoing research highlights the importance of accurate system representation, where wagons and their components are modeled as interconnected rigid or flexible bodies to capture complex interactions with the track and cargo [1,2,3]. Flexible multibody approaches, in particular, have been successfully applied to represent thin-walled car body structures, where structural dynamics interact with global vehicle motion [4]. These techniques are especially valuable when integrated with design methodologies aimed at mass reduction, structural efficiency, and dynamic integrity. Advances in simulation software, such as SIMPACK and MSC Adams, have facilitated the integration of sophisticated contact models, including elastic and quasi-elastic wheel–rail contact formulations, which are crucial for predicting derailment, wear, and ride quality [5]. Recent studies have expanded the scope of multibody models to include multiphysics phenomena. In parallel, design-oriented simulation approaches are increasingly incorporating structural optimization strategies to enhance vehicle components under combined dynamic and mechanical constraints, with applications focusing on lightweight design of car body structures and integration of composite materials [6,7,8,9]. The impact of cargo loading, particularly the position and shift in the center of gravity, has been shown to significantly affect safety, energy consumption, and wear, prompting the development of optimization strategies for loading plans and the use of simulation to assess curving performance and operational limits [10,11,12]. Experimental validation remains a cornerstone, with models being calibrated against field and laboratory data to ensure reliability [13,14,15]. The field has also seen the emergence of machine learning techniques, where digital twins and surrogate models are trained on multibody simulation outputs to enable rapid safety assessments and reduce computational costs [16]. Additionally, multibody models are now being used to support the development of self-powered sensor nodes for real-time condition monitoring, leveraging vibration energy harvesting to power essential safety sensors in freight wagons [17]. Overall, the literature demonstrates that multibody modelling not only enhances the understanding of railway vehicle dynamics but also drives innovation in safety, efficiency, and predictive maintenance, with ongoing research focusing on model integration, computational efficiency, and experimental validation to address the evolving challenges of modern rail transport [18]. Recent studies highlight the resurgence of articulated freight wagon designs, such as the Talbot Type Articulation, which use shared bogies to improve load-to-tare ratios for lightweight, bulky cargo. These designs require careful analysis of coupler strength and fatigue due to unique loading conditions [19]. The evolution of freight bogies has focused on balancing high tare-to-laden mass ratios, running stability, and curving performance. The Y25 bogie remains widely used in Europe for its cost-effectiveness, but newer designs like the 4L and SUSTRAIL bogies offer significant improvements in weight reduction, dynamic stability, and track friendliness, with the 4L bogie achieving a 15% mass reduction and 20% better stability compared to the Y25. Multi-criteria decision-making methods have been applied to optimize bogie material selection, identifying S355 and S235 steels as ideal for balancing safety, manufacturability, and cost [20]. Structural and dynamic analyses using finite element modeling are essential for validating new bogie frames under various load cases and ensuring compliance with safety standards [21]. In this context, the integration of optimization procedures with multibody simulation has proven especially effective for developing high-performance bogie structures. Recent contributions have demonstrated how topological optimization combined with sensitivity analysis can yield innovative bogie frame geometries that enhance mechanical performance while remaining compatible with multibody simulation environments [22,23]. Comparative studies of different bogie types show that rationalizing suspension parameters and adopting innovative designs can improve dynamic indicators, reduce wear, and lower derailment risk, especially on curves and under varying loads [24,25,26]. Additionally, noise reduction strategies, such as applying acoustic silencers to bogie frames, have shown promise in mitigating environmental impacts [27]. Although articulated freight wagons are increasingly adopted in European intermodal transport, their dynamic behavior has received limited attention in the scientific literature. In particular, no comprehensive study has yet been carried out on the T3000 articulated wagon, despite its widespread use across the European rail network. Existing research mainly focuses on conventional two-bogie freight wagons, providing only partial insight into the dynamic phenomena associated with articulated configurations. The absence of validated models and dedicated experimental data for such vehicles has so far limited the understanding of their complex interactions, including those between shared bogies and coupling systems. In order to address these gaps, the present work proposes a comprehensive approach combining an extensive onboard measurement campaign with high-fidelity multibody modeling. A dedicated experimental setup, including inertial sensors and a 3D vision system, was designed to capture the vehicle’s global dynamics, the interaction between adjacent wagons, and the behavior of buffer and coupler assemblies. The acquired data were used to tune and validate a detailed multibody model of the T3000 wagon, leading to the development of a robust digital twin capable of accurately reproducing the real system’s dynamic response. This research contributes to bridging the gap between experimental and numerical analysis of articulated freight wagons, paving the way for predictive maintenance strategies and advanced virtual testing. This integrated framework establishes the foundational architecture for a digital twin, where the high-fidelity virtual environment is designed to be fed by the onboard sensing layer for advanced diagnostic and monitoring applications.

## 2. Materials and Methods

### 2.1. T3000 Articulated Intermodal Freight Wagon

**Figure 1:**
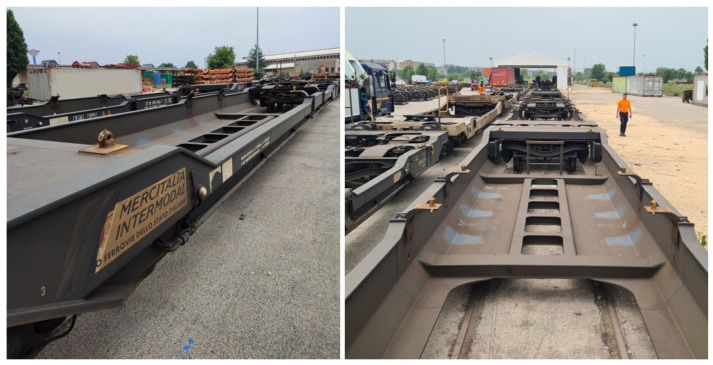
T3000 articulated freight wagon (on field views).

The T3000 is a modern articulated intermodal freight wagon specifically designed for the transport of semi-trailers and swap bodies in combined railroad operations. Widely used across the European freight network, the T3000 wagon is characterized by a distinctive architecture consisting of two half-frames connected via a central articulation, supported by three bogies: two at the ends and one in the middle. Figure 1 shows it.

This configuration allows for improved axle load distribution and higher transport capacity while maintaining compatibility with standard infrastructure limits. A key feature of the T3000 is its two-stage suspension system, which enhances ride comfort and reduces dynamic loads transmitted to the cargo. Additionally, the wagon incorporates a central pivoting turntable equipped with sliding pads that facilitate relative motion between the half-frames during curving, and articulated couplers that play a critical role in the longitudinal dynamics of the vehicle. These design features, although advantageous in terms of operation, introduce a level of complexity in dynamic modeling that goes beyond the standard freight wagon paradigm. A short summary of the main characteristics of the T3000 wagon is presented in Table 1.

**Figure 2:**
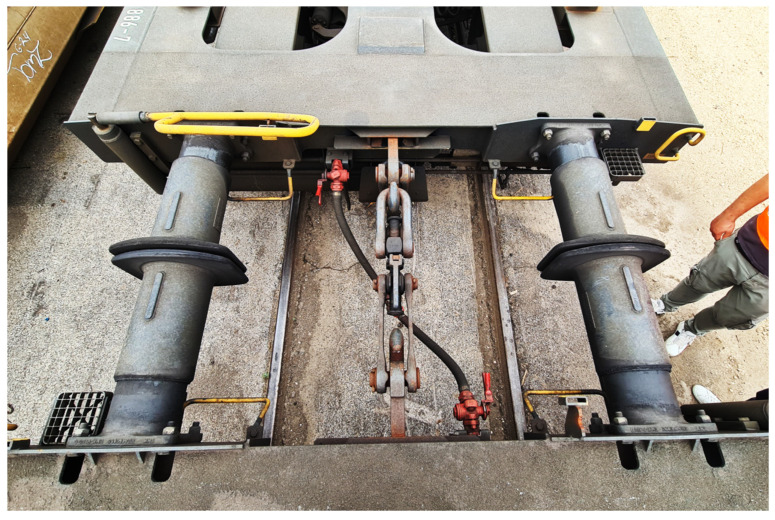
Detailed on field view of side buffers and traction equipment (draw gear and screw coupling).

The T3000 articulated wagon is equipped with three Y25-type bogies, a widely adopted standard in European freight rolling stock due to its robust design, reliability, and ease of maintenance [28,29,30,31,32]. The Y25 bogie features a two-axle configuration with a welded steel frame and is designed to carry high axle loads while ensuring acceptable dynamic performance over a wide range of operating conditions. Each bogie is equipped with a primary suspension system composed of coil springs and friction dampers (using Lenoir links), which serve to attenuate vertical and longitudinal vibrations. The wheelsets are guided transversely by swing arms, and the axle boxes are mounted with a combination of elastic and damping elements that influence the vehicle running stability, especially at higher speeds. Its modularity and standardization make it a suitable platform for integration within more complex vehicle architectures like the T3000, where one of the bogies is placed at the articulation point between the two wagon halves. The combined action of the center bowl and sliding pads introduces significant nonlinearities into the dynamic behavior of the system, particularly under combined loading scenarios. As such, this subsystem has been carefully modeled in the multibody environment to capture both its kinematic function and force-transmitting characteristics. Despite being a conventional bogie, the Y25 plays a significant role in the overall dynamic behavior of the articulated system and must be accurately represented within the multibody model. At the articulation point between two coupled wagons, the mechanical interface composed of two side buffers and the traction equipment plays a fundamental role in controlling the transmission of forces and the relative motion between the interconnected vehicles. The interface, is shown in Figure 2. The buffers, whose behavior was studied during this experimental activity, absorb compressive loads during pushing and braking phases. Typically made of steel housings with internal elastomeric or hydraulic elements [33,34,35], they provide both energy dissipation and elastic compliance, reducing impact severity and limiting the propagation of stresses through the structure, their symmetric arrangement ensures balanced force distribution and helps maintain lateral alignment during dynamic transitions. In contrast, the traction equipment, consisting of the draw gear and the screw coupling placed along the longitudinal centerline, is responsible for transmitting tensile forces during acceleration and hauling, and contributes to constraining the longitudinal relative motion between the two wagons, ensuring coordinated movement during operation. The parallel configuration of buffers and traction equipment generates a nonlinear force sharing mechanism whose response depends on the direction and magnitude of the applied loads. As part of future developments, an experimental campaign is planned to further characterize the behavior of this interface, with particular focus on the performance of the buffers.

An essential aspect of the articulation system in the T3000 wagon is the geometric interface between the two half-frames, which allows for controlled relative rotation while maintaining structural continuity and limiting undesired motion. The wagon halves are shaped in such a way that their mating ends form a specially contoured profile, optimized to accommodate relative yaw motion during curving while avoiding mechanical interference. This interface acts as a passive guiding system, ensuring that the two frames remain correctly aligned under normal operating conditions. The cut-out geometry is designed to allow a limited angular excursion about the vertical axis, thus facilitating curve negotiation without imposing excessive constraints on the articulation. At the same time, the shape and clearance of the profiles are engineered to prevent excessive yaw displacements, which could compromise dynamic stability or lead to mechanical contact under extreme loading scenarios. This careful balance between freedom of motion and geometric constraint contributes to the overall torsional and lateral stiffness of the articulated joint, enhancing both safety and dynamic performance. The role of this interface, although purely geometric in nature, is non-negligible in the multibody representation of the vehicle, and it has been properly modeled using suitable kinematic constraints.

### 2.2. Experimental Setup

**Figure 3:**
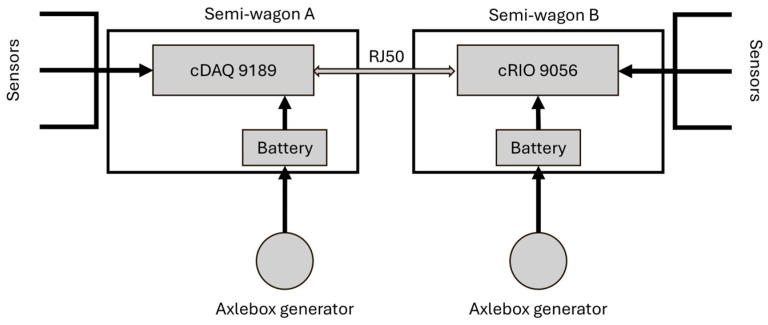
Scheme of data acquisition chain and power supply system.

In the development of a physics-based digital twin for freight wagon monitoring, defining an appropriate experimental setup is essential to ensure the reliability of the virtual model. This is particularly relevant for complex vehicles such as the T3000 articulated wagon, which has never undergone a complete physical characterization. The test setup is therefore designed to reproduce real operating conditions and provide high-quality data for model calibration and validation, enabling effective condition monitoring and supporting future homologation and maintenance applications. In this research activity, supported by an extensive experimental campaign, great attention was devoted to defining the most effective instrumentation layout to capture the dynamic behavior of the T3000 articulated freight wagon, with the objective of developing a reference digital twin of the vehicle. This digital twin aims to fill an important gap identified in the current literature, providing a comprehensive framework and practical guidelines for future studies and applications related to the experimental characterization and digital replication of freight wagons. The adopted setup combines multiple types of inertial sensors strategically positioned to monitor the running dynamics of the vehicle, a set of wire potentiometers, load cells and a dedicated 3D vision system for the detailed observation of the buffer performance. Beyond the sensors themselves, particular care was given to the design and implementation of the power supply and data acquisition chain, which was thoroughly validated and subsequently integrated on the test vehicle to ensure high reliability and measurement consistency throughout the tests. The data acquisition system was powered by two axle box generators, each charging a battery pack to ensure reliable operations. The scheme is presented in Figure 3. These generators supplied power to two National Instruments units: the cDAQ-9189 and the cRIO-9056, each mounted on one of the two semi-casings of the vehicle. The cDAQ-9189 is an 8-slot Ethernet chassis designed for modular data acquisition, while the cRIO-9056 is a rugged, real-time controller with integrated I/O capabilities, suitable for industrial applications. To enable synchronized data collection between the two units, they were interconnected and synchronized via an RJ50 connector. All sensors provided their signals to these two acquisition units, ensuring comprehensive data capture across the vehicle’s structure. The use of the RJ50 connector enabled efficient and reliable communication between the units, supporting the synchronized operation essential for accurate data analysis. All signals were sampled at a frequency of 512 Hz save from the accelerometers mounted on the axle boxes, which were sampled at a frequency of 2048 Hz to ensure accurate reproduction of high frequency behaviors.

**Figure 4:**
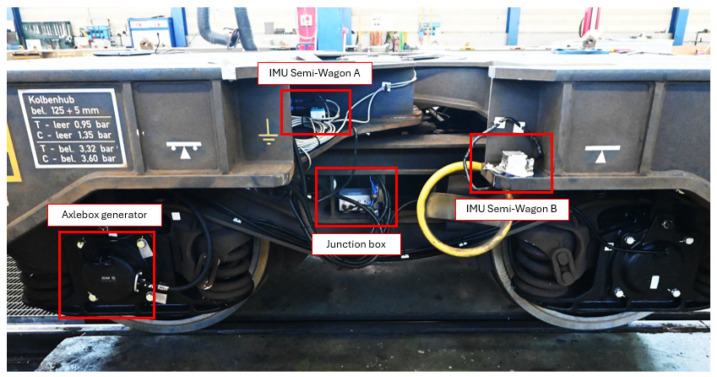
Main equipment positioning: IMUs, junction box and axle box generator.

The experimental setup employed for the dynamic characterization of the vehicle, included two MRM60IMU inertial measurement units, manufactured by Gladiator Technologies (reference number 490-15-100, Snoqualmie, WA, USA). These high-performance IMUs provide six degrees of freedom measurements, enabling the acquisition of tri-axial linear accelerations and tri-axial angular velocities with high resolution and stability. One unit was mounted on the semi-wagon A and the other half of the articulated freight wagon. This configuration was chosen to capture the relative dynamic behavior of the two sections under operational conditions, particularly during curve negotiation and transitions. The dual-sensor arrangement allows for a detailed analysis of rigid-body motions such as pitch, roll, and yaw, as well as the detection of bending modes and torsional deformations that may occur along the vehicle’s length. The MRM60IMU was particularly well-suited for railway applications due to its compact form factor, robust construction, and high-performance MEMS sensors, which provide low noise and stable measurements even under harsh vibrational environments typical of freight operations. Its wide operational temperature range and resistance to shock and vibration make it ideal for on-board installation, ensuring reliable data acquisition throughout the full duration of the tests. The precise measurement of inertial quantities at both ends of the wagon is essential for validating the multibody model and for tuning its parameters to reflect the real vehicle dynamics more accurately. The positioning of the two IMUs, the junction box and of the axle box generator, is clearly illustrated in Figure 4.

**Figure 5:**
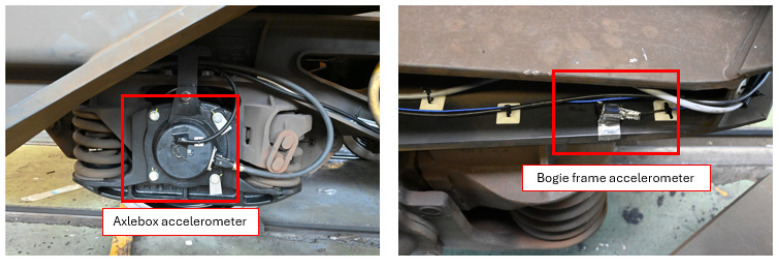
Main equipments positioning: axle box accelerometer and bogie frame accelerometer generator.

The wagon was also instrumented with accelerometers to enable a detailed analysis of its dynamic response during operation. Each of the three bogies was equipped with a consistent configuration of accelerometers comprising one uniaxial, one biaxial, and one triaxial sensor. This layout was designed to capture the full spectrum of vibrational modes affecting the bogie frame and axle box region. Specifically, the triaxial accelerometer was mounted on the bogie frame to acquire linear accelerations along the longitudinal (X), lateral (Y), and vertical (Z) directions. The biaxial and uniaxial sensors complemented these measurements by providing redundancy and increased resolution in selected directions. In addition to this standard configuration, as depicted in Figure 5, the intermediate bogie, was further instrumented with uniaxial accelerometers mounted directly on the axle boxes. These sensors were oriented to measure vertical accelerations (Z-direction), which are of particular interest for assessing wheel–rail interaction forces and detecting high-frequency phenomena such as impacts, track irregularities, and local dynamic amplifications. A detailed summary of the technical specifications and the specific placement of each sensor type is provided in Table 2.

The sampling frequencies reported in Table 2 were defined to satisfy the Nyquist-Shannon criterion for all investigated phenomena. This ensures that the signal integrity is preserved across the entire frequency range of interest, preventing aliasing artifacts in the acquisition of both the low-frequency vehicle dynamics and the high-frequency transients associated with wheel–rail interaction. The installation of sensors directly on the axle boxes enables a more localized and sensitive detection of dynamic loads transmitted through the primary suspension system, providing essential input for validating both vertical dynamics and contact models within the multibody simulation framework. The combination of frame-mounted and axle box-mounted accelerometers ensures a comprehensive characterization of the system dynamics, from global vehicle motions to localized contact-induced excitations.

**Figure 6:**
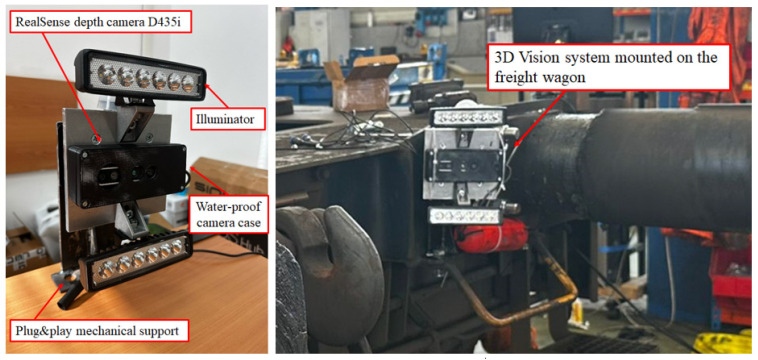
Three-dimensional vision system for buffer monitoring [36].

At the headstock of the instrumented wagon, two wire-type potentiometers, each with a measurement stroke of 150 mm and dedicated to one buffer, were installed together with a 3D vision system designed to monitor the buffers’ performance during dynamic tests. The use of wire potentiometers allowed precise tracking of the relative displacements over the entire working range of the buffers, even under high-frequency oscillations and large deformation amplitudes. This integrated instrumentation setup enabled a complete characterization of the interaction at the wagon interface, allowing the relative motion of each buffer to be accurately measured. In addition to the wire potentiometers, four HBK KMR400 load cells (Darmstadt, HE, Germany) were mounted between each buffer and the wagon headstock. Each load cell has a sensitivity of 2 mV/V at the nominal load of 400 kN. The sum of the reading of the four cells was used to estimate the longitudinal force experienced by each buffer. By combining the displacement data with the buffers’ measured longitudinal force, it was possible to estimate the forces exchanged between the two wagons during impact and compression phases, thus providing a comprehensive assessment of the mechanical behavior of the coupling system. As introduced above, in the experimental setup, an RGB-D vision system was employed to monitor the dynamic behavior of the freight wagons. The sensing unit consists of an Intel RealSense D435i camera (Santa Clara, CA, USA), selected for its ability to acquire synchronized color and depth data. For installation reasons, the camera was mounted in an inverted orientation, requiring a 180° rotation of the captured frames during the data processing stage. The recorded data, stored in a compressed *.bag* format, were processed through a dedicated Python script developed to extract the RGB and depth streams, perform their spatial and temporal alignment, and export the synchronized datasets into structured MATLAB (*.mat*) files for subsequent analysis. In this configuration, shown in Figure 6, the D435i camera acquired color and depth images with a resolution of 640 × 480 pixels at 15 frames per second, ensuring consistent temporal sampling across the entire dataset used for performance evaluation [36].

### 2.3. T3000 Multibody Model

**Figure 7:**
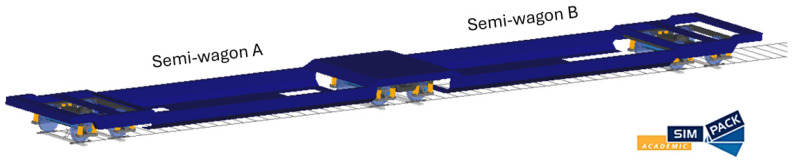
Multibody model of T3000 articulated wagon created using Simpack software.

As a core element of the proposed digital twin, a high-fidelity multibody model of the T3000 articulated freight wagon was developed in Simpack. The model was designed to reproduce the full kinematic architecture of the articulated system and to provide an accurate representation of the vehicle’s dynamic behavior under operational conditions. The T3000 articulated wagon, consists of two semi-car bodies resting on three Y25 bogies. The central bogie is shared by both semi-frames, which are mechanically connected through articulated joints. The multibody model was created starting from the original design drawings of the vehicle, from which the geometry of the two semi-car bodies was reconstructed, as shown in Figure 7. Subsequently, their mass and inertial properties were estimated and implemented in the model, as discussed in the next sections.

The bogie used in the T3000 articulated wagon is based on the Y25 design, which is a standard configuration for European freight applications. The bogie frame is characterized by a robust welded structure designed to accommodate high axle loads and ensure durability under demanding operating conditions. The primary suspension system consists of coil springs and dampers mounted between the axle boxes and the bogie frame, providing the necessary flexibility and damping to filter track-induced excitations. Uncommonly for freight wagons, the T3000 integrates additional suspension elements between the central bogie and the semi-car body, consisting of vertical springs and lateral dampers that enhance ride quality. The connection between the bogie and the car body is achieved through a center pivot, also referred to as a center bowl bearing system, which transmits vertical loads while allowing relative yaw rotation. Additionally, side bearer work with center pivot to guide the rotation and support transverse loads, contributing to stability during curve negotiation and dynamic maneuvers. Both the center bowl bearing and the sliding pads were modeled in the multibody environment using the Connection element available in Simpack. This modeling choice offers a higher degree of flexibility compared to alternative approaches such as Element Force or Joint, particularly in the context of frictional behavior. The Connection formulation enables the definition of a comprehensive set of parameters governing the relative motion between contacting components, including friction laws, limit forces, preload conditions, and friction coefficients. This level of detail is essential for accurately capturing the nonlinear and direction-dependent behavior of the interfacial forces between the car body and the bogie. In this work, the frictional interaction in both the center bowl bearing and the sliding pads was modeled assuming a dry steel-on-steel contact condition, which is representative of the physical interfaces in the investigated vehicle. The adoption of this friction model allowed the specification of a reference friction coefficient and associated dynamic parameters, leading to an improved correlation between simulation results and experimental data. This approach proved to be particularly effective in reproducing the observed behavior during curving and transition phases, where frictional moments and resistance to yaw motion play a significant role in the global dynamics of the articulated freight car. Both primary suspension system and side bearer were defined within the substructure associated with each individual bogie, using the spring-damper parallel CMP formulation. This approach is considered the most suitable for the current application, as it allows for a straightforward and accurate representation of the linear and nonlinear stiffness and damping properties of the suspension components while maintaining numerical stability and computational efficiency. The use of substructures not only facilitates a modular and scalable modeling strategy but also significantly reduces the likelihood of errors during the construction of the full vehicle model, an advantage that becomes increasingly important when dealing with articulated wagons or complex trainsets. By defining the suspension behavior within a reusable substructure, the model can replicate identical bogie components without redundancy, ensuring consistency across the system and simplifying future modifications or parameter studies. Furthermore, both the bogie frames and wheelsets were modeled as rigid bodies, with all relevant mass and inertial properties explicitly defined according to manufacturer data. In the case of the wheelsets, each one was also associated with a track element, which is required in Simpack to define the rail geometry and establish the reference trajectory for the simulation. This association ensures correct positioning and orientation of the wheel–rail contact interface throughout the vehicle’s motion, enabling accurate simulation of curving behavior, load transfer, and dynamic interactions with the track. Finally, the wheel–rail contact interface was implemented in the multibody model using the standard 60E1 rail profile and the S1002 wheel profile, a combination representative of conventional European railway infrastructure. This pairing, which characterizes the investigated line segment, was selected to accurately reproduce the contact conditions encountered during vehicle operation. The wheel–rail contact problem is addressed using Kalker’s simplified FASTSIM algorithm, a well-established approach within the Simpack Rail environment. This formulation ensures an optimal balance between physical accuracy in creep force calculation and computational efficiency, making it highly suitable for complex multibody dynamic simulations. The profiles were defined according to the specifications provided in EN 13674-1 [37] for the rail and EN 13715 [38] for the wheel, ensuring compliance with international standards. The geometric data were discretized and imported into the simulation environment. Furthermore, the selected contact profiles are consistent with those used in the experimental validation phase, allowing for direct comparison between simulation and test results, showing an optimum agreement. The complete multibody model underwent an extensive tuning phase, where key parameters, specifically primary suspension stiffness, damping coefficients, and side bearer characteristics, were iteratively refined to ensure an accurate reproduction of the overall dynamic behavior of the wagon. This calibration, guided by physical insight and component specifications, was essential to achieve the level of fidelity required for a reliable digital twin, with the resulting model performance and its detailed comparison against test-track data presented in the following section.

## 3. Results and Discussions

**Figure 8:**
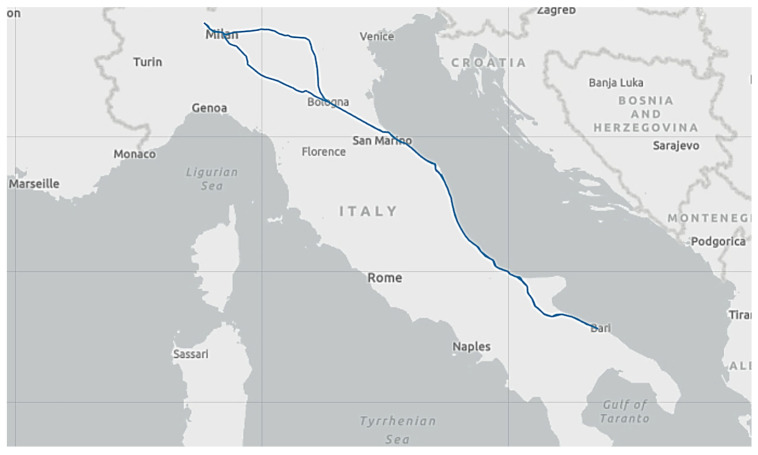
Test route covered by the T3000 articulated freight wagon during the experimental campaign along the Italian Adriatic railway line.

The instrumented T3000 freight wagon was operated within a regularly scheduled freight convoy along the Adriatic railway line, covering the route from Busto Arsizio to Bari Lamasinata, as shown in Figure 8.

This line, part of the Italian national railway network, is fully electrified at 3 kV DC and predominantly double-tracked, with mixed passenger and freight traffic. The route comprises a combination of flat and slightly undulating terrain, with long straight sections, particularly along the Adriatic coast, interspersed with curves and a limited number of gradient changes. Between Ancona and Bari, major scheduled stops included Pescara, Termoli, Foggia and Barletta, reflecting typical freight operations along the southern segment of the corridor. The overall distance covered was approximately 2000 km. The wagon, with a total mass of approximately 53 tonnes, coinciding with its braked mass, was monitored throughout the journey. During the run, the vehicle reached a maximum speed of 110 km/h on a straight, level segment of track. The results obtained from the experimental campaign are presented and discussed in comparison with numerical results derived from the dedicated multibody model of the wagon developed in Simpack, properly tuned based on the experimental data. In order to validate the experimental model and ensure its reliability, two representative track segments were selected: a curved section, used to evaluate the signals collected with the onboard IMU, and a straight section, used to study the vehicle’s hunting motion. Both analyses yielded good results, providing a thorough assessment of the numerical model’s accuracy and the experimental campaign’s potential across different track conditions. Detailed experimental results and analysis concerning the 3D vision system and buffer displacement have been extensively discussed in previous work cited above; therefore, they are omitted here to avoid redundancy and to focus on the multibody model validation.

### 3.1. Case Study: Curved Track Segment Analysis

The selected case study corresponds to a track segment characterized by a curvature of 0.002 m^−1^, equivalent to a circular curve with a radius of 500 m. The wagon traversed this segment at an approximate speed of 90 km/h, resulting in a total duration of about 37 s. The track geometry includes a straight section, followed by an entry transition curve (clothoid), a central circular arc representing the target curvature, and an exit transition curve leading back to a straight path. The cant (superelevation), included in a range between 130 mm and 150 mm, was appropriately set along the different sections of the segment to realistically reproduce the actual track conditions and to ensure the correct representation of the lateral equilibrium during curve negotiation. Table 3 sums up the track properties according to Simpack table format. In the numerical simulations, predefined power spectral densities referred to as Track Irregularity ERRI B176 were introduced to account for both vertical and lateral track irregularities. This approach ensures a realistic excitation of the vehicle–track system, as the ERRI B176 spectra are widely recognized for their ability to represent typical track conditions in dynamic studies. By incorporating these standardized irregularity profiles, the numerical model achieves high representativeness, thereby enhancing the reliability of the comparison with experimental measurements. Signal filtering was performed by applying standardized low-pass filters to remove high-frequency noise while preserving the relevant vehicle dynamic content. The selected segment thus provides a meaningful test case for evaluating the dynamic response of the wagon under moderate curvature conditions, where lateral dynamics are more pronounced than in straight-track operation. The results presented in the following refer to selected inertial sensors mounted on the instrumented T3000 wagon.

#### 3.1.1. Comparison Between Measured and Simulated Data: Semi-Wagon A—IMU

**Figure 9:**
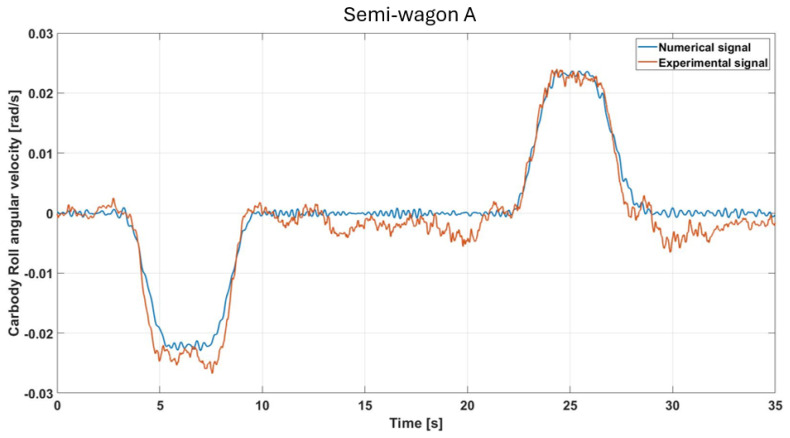
Comparison of simulated and measured roll-velocity responses under the specified test conditions (semi-wagon A).

Figure 9 shows the roll angular velocity of the wagon’s car body as measured by the IMU positioned on the semi-wagon A and compares the experimental signal with the numerical prediction from the multibody model. The numerical model accurately reproduces the main features of the experimental signal, including the roll increase in the clothoid and circular sections due to centrifugal acceleration and cant, as well as the curve entry effect followed by subsequent damping (near 5 s). The inclusion of track irregularities in the model significantly improves the agreement between experimental and numerical signals, except for occasional unpredictable fluctuations caused by the real track, such as those observed between 10 and 20 s. Overall, the model is able to reproduce the T3000 wagon’s experimental behavior accurately. More in detail, during the initial phase of the signal (approximately between 0 and 3 s), the vehicle travels along a straight track; therefore, the roll angular velocity oscillates around zero, also due to the vehicle hunting motion. When the vehicle enters the curve, in the time interval approximately between 3 and 5 s, the roll angular velocity varies with an almost linear trend within the transition curve (clothoid), where the cant actually changes from zero to the maximum value characterizing the circular section of the curve. Once the vehicle is on the circular section, the roll angular velocity remains constant, as the cant also remains fixed at a specific value, which in this case is approximately 140 mm. In order to perform a detailed comparison of the differences between the real signal and the simulated one, the value of the Root Mean Square Error (RMSE) was also evaluated. In this case, the resulting error amounts to approximately 1.46%.

**Figure 10:**
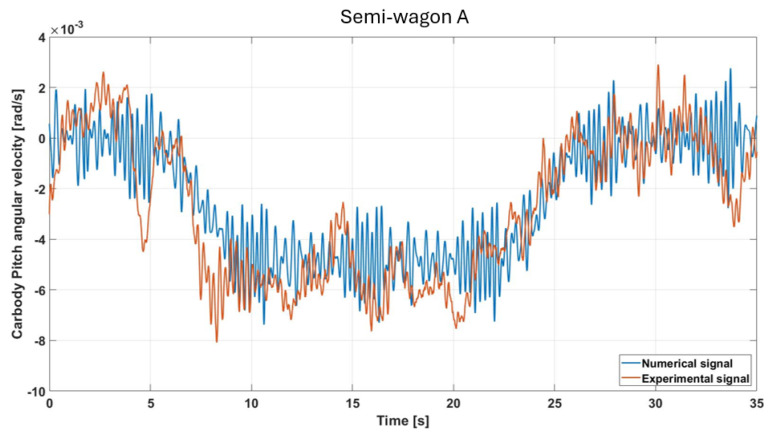
Comparison of simulated and measured pitch-velocity responses under the specified test conditions (semi-wagon A).

Figure 10 shows the pitch angular velocity measured by the same sensor. The experimental signal reveals small but noticeable pitch oscillations, generally distributed uniformly along the track. These can be attributed to vertical track irregularities, axle hunting effects, or slight inconsistencies in the vertical suspension behavior under dynamic loading. Their low magnitude, on the order of 10^−3^ rad/s, highlights the absence of impulsive dynamics that could potentially arise from car-to-car interactions. This is confirmed by the consistency between the experimentally observed oscillations and the numerical predictions, which exhibit comparable amplitudes. The pitch channel is particularly sensitive to local imperfections, such as rail joints or minor vertical dips, which can momentarily tilt the car body forward or backward. The agreement between experimental and numerical results is remarkably good. As already noted for the roll angular velocity, in this case as well the temporal variation in the pitch angular velocity is governed by the change in cant between the straight, the clothoid, and the constant-radius curve. Also in this case, the RMSE was evaluated, yielding a value of approximately 3.4%.

**Figure 11:**
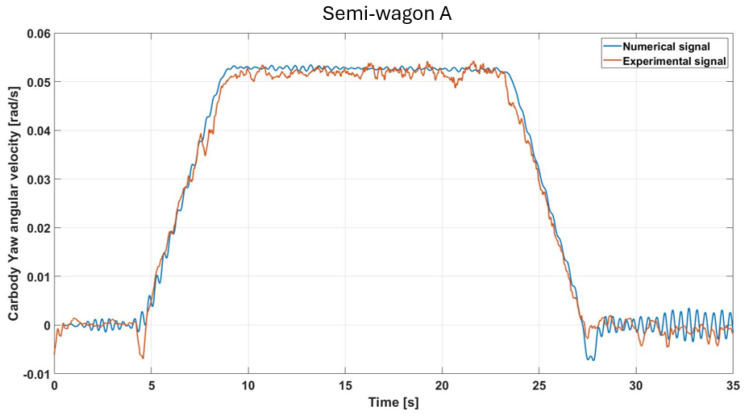
Comparison of simulated and measured yaw-velocity responses under the specified test conditions (semi-wagon A).

Figure 11 illustrates the yaw angular velocity, showing excellent agreement between the experimental and numerical signals in terms of both amplitude and temporal evolution. The profile is symmetric and reflects the expected behavior for a track with a clothoid–circular–clothoid layout. During the steady-state portion of the curve, the yaw rate remains nearly constant, corresponding to the imposed curvature and vehicle speed. Minor discrepancies in amplitude, particularly in the transition zones, may result from lateral track irregularities (such as gauge variation or local misalignments) and wheel–rail contact conditions, including flange interaction. The overall agreement supports the validity of the model in reproducing the primary yaw dynamics of the vehicle. Also in this case the evaluated RMSE amounts to approximately 3.4%.

#### 3.1.2. Comparison Between Measured and Simulated Data: Semi-Wagon B—IMU

**Figure 12:**
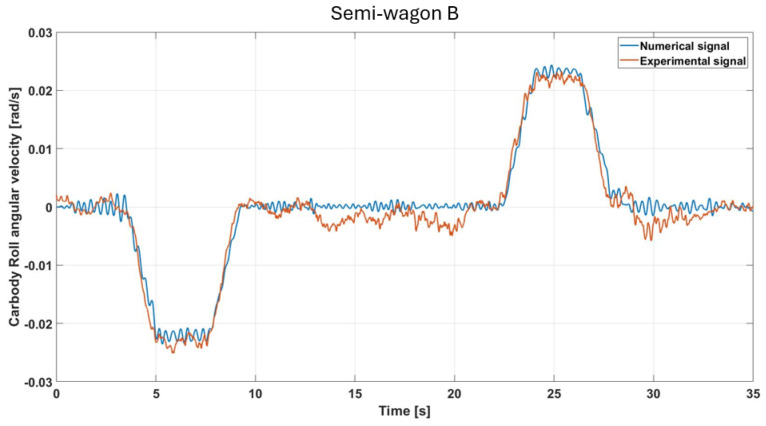
Comparison of simulated and measured roll-velocity responses under the specified test conditions (semi-wagon B).

The roll angular velocity at the rear car body exhibits a global behavior consistent with that observed at the front (Figure 9), with the primary variations occurring during the transition into and out of the curved track segment. Both the experimental and numerical signals, illustrated in Figure 12, show a marked increase in roll rate during curve negotiation, peaking under 0.03 rad/s. The comparison with the numerical results further confirms the reliability of the analysis, as experimental and simulated data exhibit an optimum overall matching, with an approximate value of the RMSE near to 1.4%.

**Figure 13:**
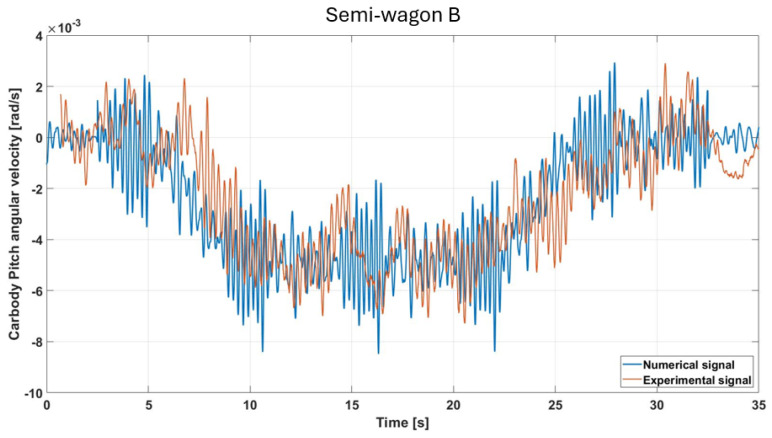
Comparison of simulated and measured pitch-velocity responses under the specified test conditions (semi-wagon B).

The pitch velocity measurements present similar characteristics to those observed in Figure 10. In this case, some peaks exceed the threshold of 8 rad/s, which is higher than in the previous case, although the values remain within the order of 10^−3^. This suggests that the rear car body of the wagon could be more sensitive to vertical perturbations. The high-frequency fluctuations may also be amplified by reflection or transmission of vertical energy along the vehicle frame, resulting in greater pitch disturbances at the rear than at the front, as shown in Figure 13.

**Figure 14:**
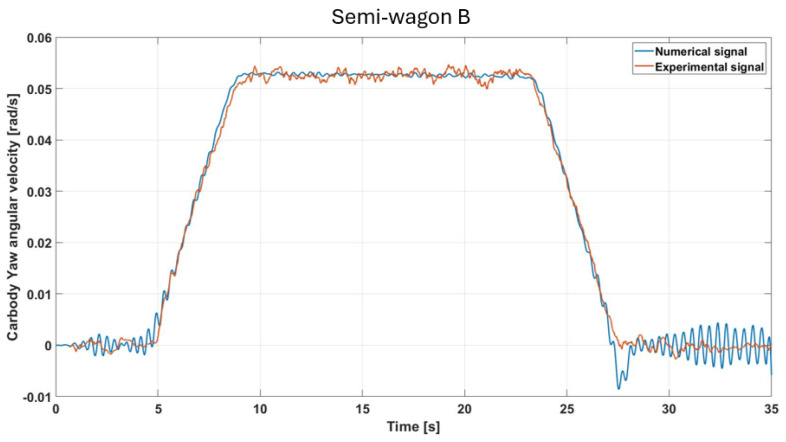
Comparison of simulated and measured yaw-velocity responses under the specified test conditions (semi-wagon B).

The yaw angular velocity displays a trend broadly consistent with the front IMU data (Figure 10), with both signals showing a steady yaw rate in the circular curve section, followed by rapid transitions during the clothoids. Figure 14 shows that the maximum pitch value, consistent across both sensors, is slightly above 0.05 rad/s. In this case, the agreement between numerical and experimental signals is excellent, further confirming the validity of the model.

Globally, the main trends and magnitudes remain consistent between the front and rear positions, confirming both the structural coherence of the wagon and the reliability of the measurement setup. The numerical model provides an accurate reference for all three axes at both locations, and the experimental data from the two IMUs show excellent mutual consistency. The strong agreement between numerical and experimental results therefore allows us to positively conclude the validation of both IMUs.

### 3.2. Case Study: Straight Track Segment Analysis

**Figure 15:**
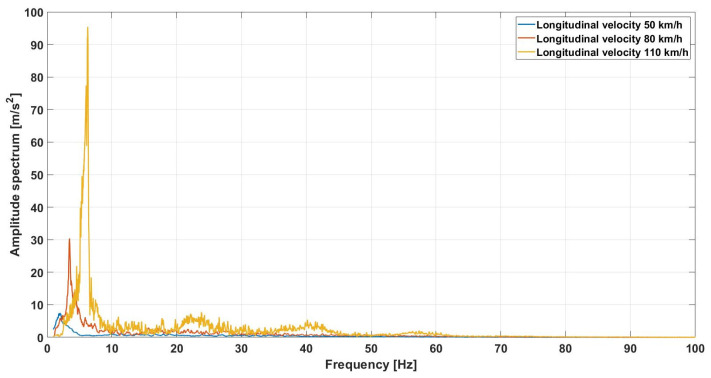
Lateral acceleration spectrum comparison: experimental hunting.

As shown in the previous sections, the vehicle is equipped with extensive onboard instrumentation, which allowed the collection of a large amount of data on various aspects of its dynamics. Of fundamental importance is the study of the vehicle’s hunting motion, which primarily involves the lateral acceleration components of the system. The signals recorded by the IMU were not sufficient to capture this motion. In contrast, the accelerometers successfully detected the presence of hunting motion. The initial analysis focused on a detailed examination of the lateral acceleration spectrum along the straight track segment at various vehicle speeds. Prior to spectral analysis, the signals were properly filtered to remove noise and unwanted components. The Fourier transform was then applied to examine the frequency content of the lateral acceleration. Figure 15 presents the spectral content of the lateral acceleration measured on the mid-bogie frame at three test speeds (50, 80, and 110 km/h). A prominent low-frequency peak is clearly observed, which progressively shifts toward higher frequencies and amplifies as the speed increases. Specifically, the dominant frequency moves from approximately 2 Hz at 50 km/h to nearly 6 Hz at 110 km/h. This frequency shift is consistent with the kinematic nature of hunting motion, where the oscillation frequency is fundamentally linked to the vehicle speed and the wheelset geometry. According to the classical Klingel’s formulation, the frequency increases linearly with velocity, a phenomenon clearly captured by the onboard accelerometers. Furthermore, the marked increase in peak amplitude at 110 km/h indicates a reduction in the dynamic damping of the system as it moves closer to its critical instability speed. The ability of the model and the instrumentation to track these variations is crucial for stability monitoring: a shift in these peaks toward specific frequency bands could excite structural resonances of the bogie or car body, leading to a significant degradation of ride quality and safety. While several higher-frequency peaks are also evident in the spectra, they relate to local structural vibrations and remain outside the scope of the current stability analysis.

**Figure 16:**
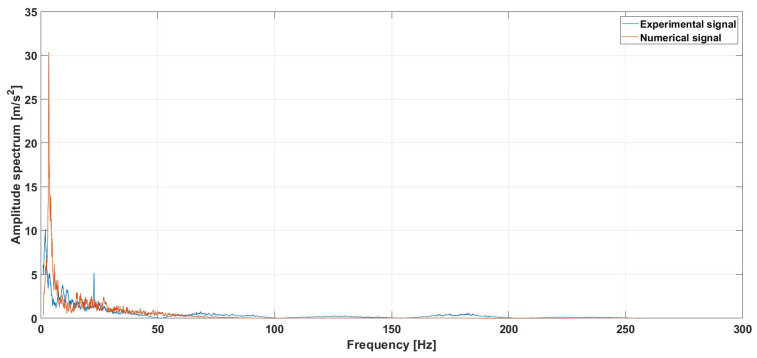
Lateral acceleration spectrum comparison for hunting analysis: experimental vs. numerical.

An additional analysis was also carried out to enable a numerical–experimental comparison specifically regarding the hunting motion, focusing in particular on the median speed of 80 km/h. Figure 16 illustrates the numerical–experimental comparison of the hunting motion at 80 km/h. The model demonstrates a high fidelity in capturing the fundamental instability mode, confirming its reliability for monitoring the vehicle’s dynamic state and identifying the dominant frequency of oscillation. As expected in a high-fidelity validation, some specific discrepancies in peak amplitude and frequency can be observed and physically justified. The larger spectral amplitude and sharper profile of the numerical results suggest a lower level of global damping compared to the physical system. This is primarily attributed to the idealization of the track as a rigid body and the simplified representation of energy dissipation in the suspension components, which in the field contribute to a broader and more attenuated response. Regarding the slight frequency offset, this deviation (approx. 1–2 Hz) is linked to the sensitivity of hunting motion to the effective wheel–rail conicity. While the numerical model employs nominal profiles, the experimental data are affected by the actual wear state of the wheels and local track gauge variations, which directly influence the kinematic wavelength of the hunting cycle. Despite these minor variations, the alignment between the two datasets remains remarkably consistent.

## 4. Conclusions

This study presented the development and experimental validation of a high-fidelity digital twin of the T3000 articulated freight wagon, integrating onboard measurements with a physics-based multibody model. In order to support and validate the numerical model, a comprehensive experimental campaign was carried out on the Italian Adriatic railway line, from Busto Arsizio to Bari Lamasinata. The measurement layout integrated inertial sensors, accelerometers, load cells, and a 3D vision system, all installed at key locations of the articulated wagon to capture its dynamic response under a wide range of operating conditions. This setup was specifically designed to provide high-resolution data suitable for direct comparison with the numerical outputs and to guide the tuning of the most sensitive model parameters. The updated comparison between simulated and measured responses was performed on both straight and curved track segments, enabling a thorough assessment of the wagon’s overall dynamic behavior. The curved segment showed excellent numerical–experimental agreement for all major car body motions, supported by high-quality experimental data collected through the two onboard IMUs. In the straight segment, the analysis focused on the vehicle’s hunting motion, which was clearly identified experimentally and successfully captured in the numerical model, resulting in very good numerical–experimental matching for this phenomenon as well. Overall, these results confirm that the combined analysis of curved and straight track sections provides a comprehensive evaluation of the T3000 vehicle dynamics and a solid basis for a reliable digital twin. The results demonstrate the capability of the proposed framework to accurately reproduce the vehicle’s dynamic behavior under real operating conditions. In conclusion, this research work establishes a scalable physics-based foundation for a comprehensive Digital Twin framework, designed to integrate the validated multibody engine with the onboard sensing layer for advanced predictive maintenance and operational monitoring. Future developments will focus on a deeper investigation of the buffer interaction through a dedicated experimental campaign, further extending the digital twin’s capability to reproduce the full dynamic behavior of articulated freight trains.

## Figures and Tables

**Table 1:** Main characteristics of the T3000 wagon.

Variable	Value
Overall length	34.20 m
Loading length	2 × 16.18 m
Distance between bogies pivots	2 × 14.20
Tare mass	35 t
Max axle load	22.5 t
Maximum payload	100 t

**Table 2:** Technical specifications and layout of the accelerometers installed on the T3000 freight wagon.

	Channel	Instrument Type	Measurement Detail	U.m.	Sampling Frequency
Front Bogie	11	Acc. PCB Piezotronics 356A25 SN LW413232 (200 g), Depew, NY, USA	X1–bogie frame	[m/s^2^]	512 Hz
	12	Acc. PCB Piezotronics 356A25 SN LW413232 (200 Depew, NY, USA g),	Y1–bogie frame	[m/s^2^]	512 Hz
	13	Acc. PCB Piezotronics 356A25 SN LW413232 (200 g), Depew, NY, USA	Z1–bogie frame	[m/s^2^]	512 Hz
	14	3AX 500 g STR 1360 SN 97160, Aliso Viejo, CA, USA	Y2–bogie frame	[m/s^2^]	512 Hz
	15	3AX 500 g STR 1360 SN 97161, Aliso Viejo, CA, USA	Z2–bogie frame	[m/s^2^]	512 Hz
	16	Acc. PCB Piezotronics 356A25 SN LW413233 (200 g), Depew, NY, USA	Z3–bogie frame	[m/s^2^]	512 Hz
Intermediate Bogie	17	Acc. PCB Piezotronics 356A25 SN LW413648 (200 g), Depew, NY, USA	X1–bogie frame	[m/s^2^]	512 Hz
	18	Acc. PCB Piezotronics 356A25 SN LW413648 (200 g), Depew, NY, USA	Y1–bogie frame	[m/s^2^]	512 Hz
	19	Acc. PCB Piezotronics 356A25 SN LW413648 (200 g), Depew, NY, USA	Z1–bogie frame	[m/s^2^]	512 Hz
	20	Acc. PCB Piezotronics 356A25 SN LW413647 (200 g), Depew, NY, USA	Y2–bogie frame	[m/s^2^]	512 Hz
	21	Acc. PCB Piezotronics 356A25 SN LW413647 (200 g, Depew, NY, USA)	Z2–bogie frame	[m/s^2^]	512 Hz
	22	monoaxial 50g LW 67400, Depew, NY, USA	Z3–bogie frame	[m/s^2^]	512 Hz
	57	Acc. PCB Piezotronics 356A33 SN LW409152 (500 g), Depew, NY, USA	Z1-axlebox	[m/s^2^]	2048 Hz
	58	Acc. PCB Piezotronics 356A33 SN LW409152 (500 g), Depew, NY, USA	Z2-axlebox	[m/s^2^]	2048 Hz
	59	Acc. PCB Piezotronics 356A33 SN LW409152 (500 g), Depew, NY, USA	Z3-axlebox	[m/s^2^]	2048 Hz
	60	Acc. PCB Piezotronics 356A33 SN LW409152 (500 g), Depew, NY, USA	Z4-axlebox	[m/s^2^]	2048 Hz
Rear Bogie	48	Acc. PCB Piezotronics 356A25 SN LW413648 (200 g), Depew, NY, USA	X1–bogie frame	[m/s^2^]	512 Hz
	49	Acc. PCB Piezotronics 356A25 SN LW413648 (200 g), Depew, NY, USA	Y1–bogie frame	[m/s^2^]	512 Hz
	50	Acc. PCB Piezotronics 356A25 SN LW413648 (200 g), Depew, NY, USA	Z1–bogie frame	[m/s^2^]	512 Hz
	51	Acc. PCB Piezotronics 356A25 SN LW413648 (200 g), Depew, NY, USA	Y2–bogie frame	[m/s^2^]	512 Hz
	52	Acc. PCB Piezotronics 356A25 SN LW413648 (200 g), Depew, NY, USA	Z2–bogie frame	[m/s^2^]	512 Hz
	53	monoaxial 50 g LN 49834, Depew, NY, USA	Z3–bogie frame	[m/s^2^]	512 Hz

**Table 3:** Curved track segment properties (Simpack parameters).

Section Type	Description	Parameter 1	Description	Parameter 2	Description	Parameter 3
Straight	Length	130 m				
Clothoid	Length	110 m	Inlet radius	0 m	Outlet radius	500 m
Circular	Length	390 m	Inlet radius	500 m		
Clothoid	Length	100 m	Inlet radius	500 m	Outlet radius	0 m
Circular	Length	10 m	Inlet radius	−3000 m		
Clothoid	Length	10 m	Inlet radius	−3000 m	Outlet radius	0 m
Straight	Length	280 m				

## Data Availability

The original contributions presented in this study are included in the article. Further inquiries can be directed to the corresponding author.
